# Evaluation of Cell Surface Vimentin Positive Circulating Tumor Cells as a Diagnostic Biomarker for Lung Cancer

**DOI:** 10.3389/fonc.2021.672687

**Published:** 2021-05-14

**Authors:** Xiaohong Xie, Liqiang Wang, Xinni Wang, Wan-Hung Fan, Yinyin Qin, Xinqing Lin, Zhanhong Xie, Ming Liu, Ming Ouyang, Shiyue Li, Chengzhi Zhou

**Affiliations:** ^1^ Department of Pulmonary and Critical Care Medicine, Guangzhou Institute of Respiratory Health, State Key Laboratory of Respiratory Disease, National Clinical Research Center for Respiratory Disease, The First Affiliated Hospital of Guangzhou Medical University, Guangzhou, China; ^2^ Department of Clinical Medical Affairs, Hangzhou Watson Biotech, Hangzhou, China

**Keywords:** circulating tumor cells, cell surface vimentin (CSV), NSCLC, cancer diagnosis, serum tumor markers

## Abstract

**Background:**

Circulating tumor cells (CTCs) represent a collection of heterogeneous cells. Studies have shown epithelial CTCs and folate receptor (FR) positive CTCs could be used as diagnostic biomarkers for lung cancer (LC). This study aimed to determine whether cell surface vimentin (CSV) positive CTCs could be used as a biomarker for LC as well.

**Methods:**

78 treatment-naïve non-small-cell lung cancer (NSCLC) patients, 21 patients with benign lung diseases (BLD) and 9 healthy donors (HD) were enrolled in this study. CTC detection was performed using CytoSorter^®^ mesenchymal CTC kit (CSV). The correlation between CSV positive CTCs (CSV-CTCs) and LC patients’ clinicopathological characteristics would be evaluated, and diagnostic performances of CSV-CTCs and serum tumor markers for LC would be compared.

**Results:**

CTC detection rates (average CTC count: range) in LC patients, patients with BLD and HD were 83.33% (2.47: 0-8), 47.62% (0.5: 0-3) and 0% (0: 0), respectively. CSV-CTCs could be used to differentiate LC patients from the patients with BLD and HD (*P* < 0.0001). CSV-CTCs were correlated with cancer stage, lymph node involvement and distant metastasis (*P* = 0.0062, 0.0014 and 0.0021, respectively). With a CTC cut-off value of 2, CSV-CTCs would have a sensitivity and specificity of 0.67 and 0.87, respectively, for diagnosing LC. CSV-CTC positive rates showed statistical differences among HD, BLD patients and LC patients at different cancer stages (*P* < 0.0001). Furthermore, CSV-CTC positive rates were positively correlated with tumor size, lymph node involvement and distant metastasis (*P* = 0.0163, 0.0196 and 0.03, respectively). CSV-CTCs had a better diagnostic performance than serum tumor makers, such as carcinoembryonic antigen (CEA), neuron-specific enolase (NSE), cancer antigen 125 (CA125) and CA153.

**Conclusion:**

When CTC cut-off is set to 2 CTCs per 7.5 mL of blood, CSV-CTCs can be considered as an acceptable biomarker for diagnosing LC with a sensitivity and specificity of 0.67 and 0.87, respectively.

## Introduction

Lung cancer (LC) is the most common cancer and the leading cause of cancer-related deaths both worldwide and in China (1, 2). There were approximately 787,000 newly diagnosed cases and 631,000 deaths for LC in 2015 in China (3). China occupies only about 20% of the world’s population, but more than one third of the LC cases are in China. Although diagnostic and treatment modalities for LC have an enormous progress in recent years, most LC patients still have a poor prognosis with a 5‐year survival rate ranging from 4-17% depending on cancer stage and regional differences (4). “Early detection, early treatment” means that patients would have a better treatment strategy and survival outcome if the tumors were diagnosed earlier (5). However, most LC patients are already in advanced stages of disease at the time of diagnosis. Thus, early detection of LC is important to improve the overall survival.

The primary approaches to diagnose LC include medical imaging examination, serum tumor markers test and biopsy. Tumors are usually quite small at an early stage and therefore they can be hardly detected by the imaging techniques due to the sensitivity limitation. Biopsy is the gold standard for cancer diagnosis, but it cannot be performed regularly due to the invasive nature, thus it cannot be used as a surveillance means to monitor the real-time progression of disease. Serum tumor makers, such as neuron‐specific enolase (NSE), carcinoembryonic antigen (CEA), cancer antigen 125 (CA125) and CA153, are frequently used in practice for LC diagnosis (6). However, serum tumor makers generate easily false positive results due to inflammations, infections, pregnancy, or other physical conditions, rendering these markers not very trustworthy. As a consequence, to improve the clinical outcomes of LC patients, it is in need to find a reliable biomarker for better screening and early diagnosis of LC.

Circulating tumor cells (CTCs) are tumor cells that have detached from the primary tumor or metastatic lesions and escaped into circulation. CTCs can colonize other organ to give rise to a new metastatic lesion once they find a suitable site (7). Studies have suggested that CTCs represent the undergoing process of metastasis and can be used as a prognostic marker to predict clinical outcomes of LC patients(8–11). CTCs can be detected in the blood even when the tumor is clinically undetectable (typically < 0.01 cm^3^) (12), implying that CTC is a good biomarker candidate for early diagnosis of LC.

CTCs are a collection of heterogeneous cells, indicating that each CTC may differ from each other in term of cell size, gene mutation and protein expression (13, 14). Several studies have suggested the use of CTCs for screening and early diagnosis of LC (15–17). Duan et al. used GILUPI CellCollector to detect CTCs in 44 patients suspected of LC and in 20 healthy donors (HD). With a CTC cut-off of 1, CellCollector has a sensitivity and specificity of 0.53 and 0.9, respectively (15). Li et al. used a negative enrichment‐fluorescence *in situ* hybridization (NE‐FISH) method to detect CTC in 174 LC patients and 90 control and discovered that NE‐FISH had a sensitivity and specificity of 0.68 and 1, respectively (16). Chen et al. used ligand-targeted polymerase chain reaction (LT-PCR) technique to detect folate receptor (FR) positive CTCs in 756 participants, including 473 patients with non-small cell lung cancer (NSCLC), 227 patients with benign lung diseases (BLD), and 56 HD, and found that with a sensitivity and specificity of 0.76 and 0.88, respectively, FR positive CTCs could be used as a biomarker in the diagnosis of NSCLC (17).

Both epithelial and FR positive CTCs can be used as biomarkers for LC diagnosis (15–17), we would like to know whether cell surface vimentin (CSV) positive CTCs (CSV-CTCs) can be used as a biomarker for LC as well. 78 NSCLC patients, 21 patients with BLD and 9 HD were recruited in this study. The diagnostic performances of CSV-CTCs and serum tumor markers for LC would be compared and the correlation between CSV-CTCs and LC patients’ clinicopathological characteristics would be analyzed.

## Materials and Methods

### Ethics and Participants

In total, 78 NSCLC patients, including 30 stage I, 7 stage II, 13 stage III and 28 stage IV, 21 patients with BLD and 9 HD were enrolled in the First Affiliated Hospital of Guangzhou Medical University. The included patients were diagnosed between May 2019 and October 2019. All included patients had negative history of malignancy within 5 years prior to enrollment, and were treatment-naïve before enrollment. Recruited patients with BLD suffered from hamartoma, papilloma, granulomatous inflammation, fibroma, benign nodule and other lung infections. All included HD had no abnormal finding in medical imaging examination and no medical history of any malignant disease. The LC patient demographics and clinical information, including age, gender, smoking history, tumor histology, TNM stage, and serum levels of NSE, CEA, CA125 and CA153 were collected.

### Identification of CSV-CTCs

CytoSorter^®^ (Hangzhou Watson Biotech, Hangzhou, China) CSV mesenchymal CTC kit was used for CTC detection. CTC detection procedure was following the manufacturer protocol and was described in the previous study (18). In brief, the streptavidin-functionalized CytoChipNano was first coated with biotin-labeled CSV antibody before placing onto CytoSorter^®^. 7.5 mL of collected peripheral blood was first proceeded to gradient-centrifuge within 6 hours after collection to collect the peripheral blood mononuclear cells (PBMC). PBMC sample solution was then transferred into the spiral sample tube on CytoSorter^®^. The enrichment procedure was controlled by CytoSorter^®^ software designed for each CTC capturing antibody. Once the CTC enrichment was finished, the CytoChipNano was removed from CytoSorter^®^ and proceeded to the manual immunofluorescence staining of CSV-FITC (fluorescein isothiocyanate), CD45-PE (lymphocyte antigen-phycoerythrin) and DAPI (4,6-diamidino-2-phenylindole). An OPPNO immunofluorescence microscope (DSY5000X, OPPNO, Chongqing, China) was used to identify CTCs by searching for CSV-FITC positive, CD45-PE negative, and DAPI positive cells. CSV-CTCs appeared green and blue, but not orange, while white blood cells appeared orange and blue, but not green, under florescent microscope. All identified cells must be checked for morphology under bright-field.

### Measurement of Serum Tumor Markers

A 3 mL of fasting venous blood sample was collected from each patient and HD in the morning. Serum was separated by centrifugation at 4000 rpm for 10 minutes within 2 hours after blood collection. NSE, CEA, CA125 and CA153 were detected by an automatic electrochemical luminescence analyzer (Cobas e602, Roche, Germany). All serum tumor marker tests were conducted according to instrument operating manuals. 17.5 ng/mL, 5 ng/mL, 35 U/mL and 25 U/mL were considered as the upper limits of normality for NSE CEA, CA125 and CA153, respectively.

### Statistical Analysis

Statistical analyses were performed using Prism 6.0 (Graphpad, La Jolla, CA, USA) and SPSS 20 (IBM Corp., Armonk, NY, USA). The x^2^ test and Fisher’s exact test were used for the comparison of categorical parameters. One-way analysis of variance (ANOVA) was performed to calculate the differences among multiple groups. Student t test was used for continuous variables, as appropriate. The diagnostic performance was evaluated by the receiver operating characteristic (ROC) curve according to the value of sensitivity, specificity and area under curve (AUC). CTC cut-off value was determined by the highest Youden index (sensitivity + specificity - 1). Comparison of diagnostic potency of different systems was rated by the AUC value. A two-sided p value less than 0.05 was considered statistically significant.

## Results

### Detection of CSV-CTCs in LC Patients, Patients With BLD and HD

**Figure 1:**
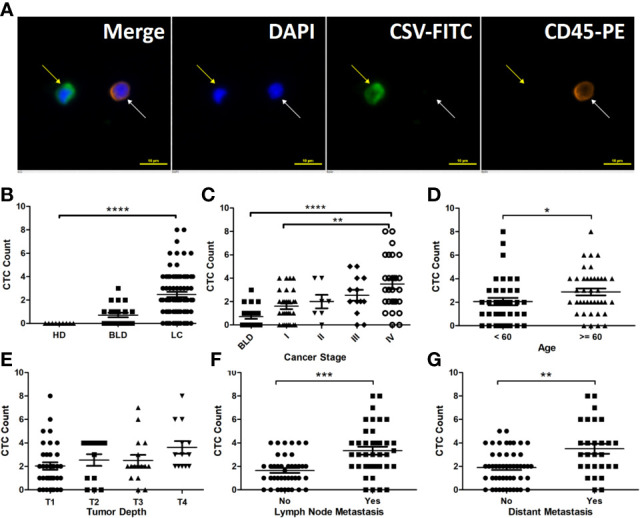
CSV-CTCs are correlated with LC patients ’ cancer stages, lymph node and distant metastases and can be used to distinguish LC patients from patients with BLD and HD. **(A)** Immunofluorescent staining of a captured CSV-CTC and a white blood cell (WBC), indicated by the yellow and white arrows, respectively. CSV positive CTCs are defined as DAPI (blue) positive, CSV (FITC, green) positive and CD45 (PE, orange) negative cells, while a WBC as a DAPI positive, CD45 positive and CSV negative cell. Scale bar represents 10 μm, immunofluorescent staining, X 20 **(B)** CSV-CTC enumeration can differentiate LC patients from BLD patients and HD (both *P* < 0.0001). **(C)** CSV-CTCs are correlated with cancer stage (*P* = 0.0062). Significant differences of CSV-CTCs are found between BLD patients and stage I, II, III or IV LC patients (*P* = 0.0167, 0.0307, 0.0014, or < 0.0001). CSV-CTCs are correlated as well with age (*P* = 0.0274), lymph node metastasis (*P* = 0.0002) and distant metastasis (*P* = 0.0021), as shown in **(D, F, G)**, respectively. However, CSV-CTCs are not associated with tumor depth as shown in **(E)**. “*”, “**”, “***”, and “****” indicates 0.01 < P < 0.05, 0.001 < P < 0.01, 0.0001 < P < 0.001, and P < 0.0001, respectively.

A CSV-CTC is shown in **Figure 1A** as a cell appearing green and blue, but not orange under florescent microscope. CSV-CTCs were detected in 65 out of 78 LC patients with a mean of 2.47 cells (range:0-8), 10 out of 21 patients with BLD with a mean of 0.5 cells (range: 0-3) and in none of the 9 HD as shown in **Table 1**. A significant difference of CSV-CTCs was found among LC patients, patients with BLD and HD (*P* < 0.0001, **Figure 1B**). Furthermore, if LC patients were broken-down by stage, significant differences of CSV-CTCs were still found between BLD patients and stage I, II, III or IV LC patients (*P* = 0.0167, 0.0307, 0.0014, or < 0.0001, **Figure 1C**), indicating that CSV-CTCs could be used as a biomarker to distinguish LC patients from the patients with BLD and HD. 9 out of 10 patients with BLD who were found to have CSV positive cells had either inflammation diseases, fibrosis or other lung infection conditions.

**Table 1:** CTCs can be used to distinguish lung cancer patients from the control (HD + BLD patients).

Group	n	Average (Median) Age (years)	CTCs ≥ 1 (per 7.5 mL)	CTC Detection Rate (%)	Average CTC Count (Range)	*P*
HD	9	58.78 (61)	0	0	0	**<0.0001**
BLD	21	51.57 (55)	10	47.62	0.5 (0-3)	
LC	78	58.55 (60)	65	83.33	2.47 (0-8)	
HD	9	58.78 (61)	0	0	0	**<0.0001**
BLD	21	51.57 (55)	10	47.62	0.5 (0-3)	
TNM I	30	55.17 (55.5)	22	73.33	1.6 (0-4)	
TNM II	7	60.29 (60)	6	85.71	2 (0-4)	
TNM III	13	61.38 (63)	11	84.62	2.54 (0-5)	
TNM IV	28	60.43 (61.5)	26	92.86	3.5 (0-8)	

CTCs, circulating tumor cells; HD, healthy donor; BLD, benign lung disease; n, number; LC, lung cancer; TNM, tumor-node-metastasis.

Bold values mean statistically significant. Bold values are all less than 0.05.

### CSV-CTCs Are Associated With Lymph Node and Distant Metastases in LC

CSV-CTC enumeration in stage I-IV LC patients ranged from 0 to 4 CTCs per 7.5 mL of blood (mean: 1.6), 0-4 (2), 0-5 (2.54) and 0-8 (3.5), respectively (*P* = 0.0062, **Figure 1C**). CTC detection rates were 73.33%, 85.71%, 84.62% and 92.86%, respectively. CSV-CTCs are positively correlated with cancer stage. Patients aged over 60 years old had a slightly higher CTC detection rate (90% compared to 76.32%) and more CTCs (2.88 compared to 2.05) than patients younger than 60 years old (*P* = 0.0274, **Figure 1D**), which might be due to that the former had usually advanced tumors. CSV-CTCs are statistically associated with lymph node involvement and distant metastasis (*P* = 0.0014 and 0.0021,respectively, **Table 2**). Patients with lymph node or distant metastasis had more CTCs (3.34 versus 1.65 or 3.5 *versus* 1.9, **Figures 1F, G**). No significant difference of CSV-CTCs was found among LC patients grouped by gender, smoking history or tumor type (adenocarcinoma or squamous) as shown in **Table 2**. CSV-CTCs were not associated with tumor depth (*P* = 0.0646, **Figure 1E**). Taken together, our results show that CSV-CTCs are correlated with lymph node and distant metastases, suggesting that the CSV-CTCs represent the CTC sub-population with more invasive nature.

**Table 2:** Correlation of CTCs with LC patients’ demographics and clinical characteristics.

Characteristics	n	Average (Median) Age (years)	CTCs ≥ 1 (per 7.5 mL)	CTC Detection Rate (%)	Average CTC Count (Range)	*P*
***Gender***						
Male	43	59.4 (60)	35	81.4	2.56 (0-8)	0.7398
Female	35	57.51 (59)	30	85.71	2.37 (0-7)	
***Age***						
≥ 60	40	65.15 (65)	36	90	2.88 (0-8)	**0.0274**
< 60	38	51.61 (52)	29	76.32	2.05 (0-8)	
***Smoking History***						
Yes	35	60.4 (60)	29	82.86	2.63 (0-8)	0.3498
No	43	57.05 (58)	36	83.72	2.35 (0-8)	
***Histology***						
Adenocarcinoma	65	58.63 (60)	55	84.62	2.42 (0-8)	0.5576
Squamous	13	58.15 (58)	10	76.92	2.77 (0-8)	
***TNM Stage***						
I	30	55.17 (55.5)	22	73.33	1.6 (0-4)	**0.0062**
II	7	60.29 (60)	6	85.71	2 (0-4)	
III	13	61.38 (63)	11	84.62	2.54 (0-5)	
IV	28	60.43 (61.5)	26	92.86	3.5 (0-8)	
***Tumor Depth***						
T1	36	55.83 (56)	28	77.78	1.37 (0-8)	0.0646
T2	13	59.92 (61)	10	76.92	2.54 (0-4)	
T3	16	59.81 (60)	14	87.5	2.5 (0-7)	
T4	13	63.15 (64)	13	100	3.62 (2-8)	
***Lymph Node Involvement***						
N0	40	56.88 (56.5)	31	77.5	1.65 (0-4)	**0.0014**
N1	4	61.5 (62.5)	4	100	4.5 (3-6)	
N2	19	61.89 (63)	17	89.47	3.26 (0-8)	
N3	15	58 (60)	13	86.67	3.13 (0-8)	
***Lymph Node Metastasis***						
Yes	38	60.32 (61)	34	89.5	3.34 (0-8)	**0.0002**
No	40	56.88 (56.5)	31	77.5	1.65 (0-4)	
***Distant Metastasis***						
M0	50	57.5 (57.5)	39	78	1.9 (0-5)	**0.0021**
M1	28	60.43 (61.5)	26	92.86	3.5 (0-8)	

CTCs, circulating tumor cells; n, number; LC, lung cancer; TNM, tumor-node-metastasis. Bold values mean statistically significant. Bold values are all less than 0.05.

### Evaluation of Diagnostic Performance of CSV-CTCs for LC

**Figure 2:**
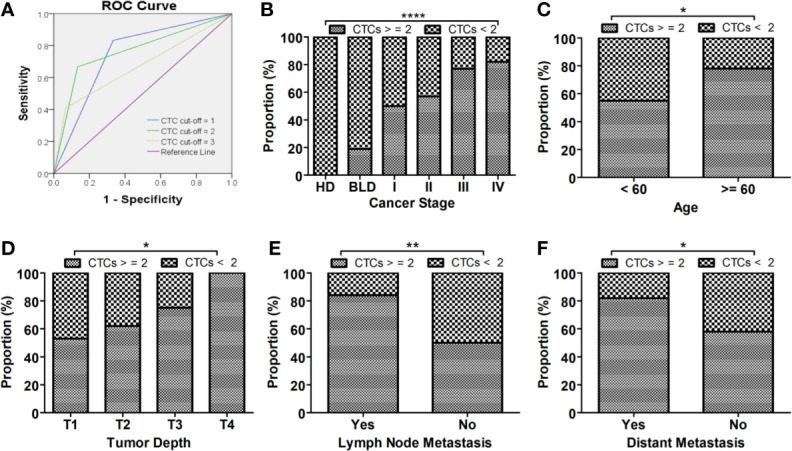
CSV-CTCs can be used as biomarker for diagnosing LC. **(A)** ROC curves of CSV-CTCs for LC with different CTC cut-off. When CTC cut-off value is set to 2, the ROC curve has the highest AUC of 0.767 with a sensitivity and specificity of 0.67 and 0.87, respectively. **(B)** CSV-CTC positive rates show significant differences among LC patients, patients with BLD and HD (*P* < 0.0001). CSV-CTC positive rates show significant differences as well between BLD and stage I, III or IV LC patients (*P* = 0.0389, 0.0014 or < 0.0001). CSV-CTC positive rates are associated with LC patients’ age, tumor depth, lymph node and distant metastases (*P* = 0.037, 0.0163, 0.0196 and 0.0013, respectively) as shown in **(C–F)**. “*”, “**”, and “****” indicates 0.01 < P < 0.05, 0.001 < P < 0.01, and P < 0.0001, respectively.

CSV-CTCs were detected in almost half of the enrolled patients with BLD (47.62%). In order to reduce the false positives in BLD patients, a ROC curve was drawn and Youden index was calculated as shown in **Figure 2** and **Table 3** to determine the CTC cut-off at which CSV-CTCs would have the best diagnostic performance for LC. When CTC cut-off was set to 1 or 2, it generated a sensitivity and specificity of 0.83 and 0.67, or 0.67 and 0.87, respectively (**Table 3**). Youden index of CTC cut-off of 2 is slightly higher than that of 1 (0.53 *versus* 0.5, **Table 3**). If CTC positive was defined as any patients with CTCs no less than 2, CSV-CTC positive rates showed a significant difference among LC patients, patients with BLD and HD as shown in **Table 4** (*P* < 0.0001) and **Figure 2B**. If LC patients were broken-down by stage, CSV-CTC positive rates showed significant differences between BLD and stage I, III or IV LC patients (*P* = 0.0389, 0.0014 or < 0.0001, **Figure 2B**). However, the CSV-CTC positive rate did not show a significant difference between BLD and stage II LC patients (*P* = 0.1423), which might be due to the small sample size of enrolled stage II patients (n = 7).

**Table 3:** Youden index of different CTC Cut-off values.

CTC cut-off (per 7.5 mL)	Sensitivity	Specificity	Youden index	Area Under Curve (AUC)
1	0.83	0.67	0.5	0.75
2	0.67	0.87	0.53	0.767
3	0.41	0.97	0.38	0.672

CTC, circulating tumor cell.

**Table 4:** CTCs can be used to distinguish LC patients from the control (BLD patients + HD) when CTC cut-off is set to 2.

Group	n	CTCs ≥ 2 (per 7.5 mL)		CTCs < 2 (per 7.5 mL)		P
		n	Proportion (%)	n	Proportion (%)	
HD	9	0	0.00	9	100.00	**<0.0001**
BLD	21	4	19.05	17	80.95	
LC	78	52	66.67	26	33.33	
HD	9	0	0.00	9	100.00	**<0.0001**
BLD	21	4	19.05	17	80.95	
TNM I	30	15	50.00	15	50.00	
TNM II	7	4	57.14	3	42.86	
TNM III	13	10	76.92	3	23.08	
TNM IV	28	23	82.14	5	17.86	

CTCs, circulating tumor cells; LC, lung cancer; HD, healthy donor; BLD, benign lung disease; n, number; TNM, tumor-node-metastasis.

Bold values mean statistically significant. Bold values are all less than 0.05.

### Correlation of CSV-CTC Positive Rates With LC Patients’ Clinicopathological Characteristics

CSV-CTC positive rates were correlated with age, lymph node involvement, lymph node and distant metastases (*P* = 0.037, 0.0196, 0.0013 and 0.03, respectively, **Table 5** and **Figures 2C**, **E, F**). CTC positive rates were not correlated with gender, smoking history, nor tumor histologic type as shown in **Table 5**. CTC positive rates was not associated with cancer stages (*P* = 0.0533, **Table 5**), although CTC positive rates did increase in more advanced LC. It might be due to the small sample size. CSV-CTC positive rate was correlated with tumor depth (*P* = 0.0163, **Table 5**).

**Table 5:** Relationship of CTCs with LC patients’ demographics and clinical characteristics when CTC cut-off is set to 2.

Characteristics	N (Total = 78)	Proportion (%)	CTCs ≥ 2 (per 7.5 mL)	CTCs < 2 (per 7.5 mL)	*P*
***Gender***					
Male	43	55.13	29	14	0.8719
Female	35	44.87	23	12	
***Age***					
≥60	40	51.28	31	9	**0.037**
<60	38	48.72	21	17	
***Smoking History***					
Yes	35	44.87	26	9	0.1979
No	43	55.13	26	17	
***Histology***					
Adenocarcinoma	65	83.33	42	23	0.3903
Squamous	13	16.67	10	3	
***TNM Stage***					
I	30	38.46	15	15	0.0533
II	7	8.97	4	3	
III	13	16.67	10	3	
IV	28	35.90	23	5	
***Tumor Depth***					
T1	36	46.15	19	17	**0.0163**
T2	13	16.67	8	5	
T3	16	20.51	12	4	
T4	13	16.67	13	0	
***Lymph Node Involvement***					
N0	40	51.28	20	20	**0.0196**
N1	4	5.13	4	0	
N2	19	24.36	16	3	
N3	15	19.23	12	3	
***Lymph Node Metastasis***					
Yes	38	48.72	32	6	**0.0013**
No	40	51.28	20	20	
***Distant Metastasis***					
M0	50	64.10	29	21	**0.03**
M1	28	35.90	23	5	

CTCs, circulating tumor cells; n, number; LC, lung cancer; TNM, tumor-node-metastasis. Bold values mean statistically significant. Bold values are all less than 0.05.

### Comparison of CSV-CTCs and Serum Tumor Markers for Diagnosing LC

**Figure 3:**
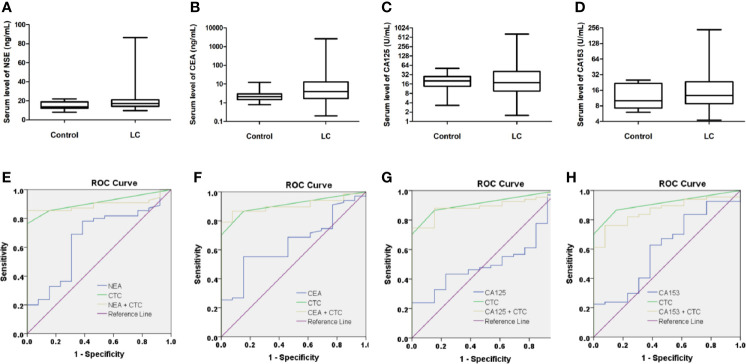
Serum levels of tumor biomarkers in control (BLD + HD) and LC patients. Serum levels of neuron-specific enolase (NSE), carcinoembryonic antigen (CEA), cancer antigen 125 (CA125) and CA153 in control and LC patients are shown in **(A-D)**. None of these serum biomarkers showed statistical significance in differentiating LC patients from the control. (box plot with mean, min to max). Conjugation of CTCs with serum markers did not improve the diagnostic performances for LC, since all AUC of combinations of CTCs with serum tumor markers were reduced as compared to CTCs alone as shown in **(E-H)**.

67 LC patients and 13 control donors (9 patients with BLD and 4HD) had serum tumor marker tests data at enrollment. Serum levels of tumor markers, including NSE, CEA, CA125, and CA153, in LC patients and control are shown in **Figures 3A–D**. No statistical difference of serum tumor marker level between LC patients and control was found, indicating that serum tumor markers were not good biomarkers for LC. However, when we compared serum tumor markers with LC patients’ clinicopathological characteristics, most serum tumor makers were correlated with cancer stage, tumor size, lymph node involvement and distant metastasis as shown in **Table 6**. Most serum tumor markers were not associated with gender, age, smoking history or tumor histologic type, except for CA153, which was correlated with age and tumor type (*P* = 0.0291 and 0.0081, respectively, **Table 6**). Serum tumor markers could reflect tumor burden in LC. LC patients at advanced cancer stage, with bigger tumors, lymph node or distant metastases, tend to have higher serum level of tumor markers. Serum tumor markers are associated as well with CSV-CTC status as shown in **Table 6**. Significant differences of NSE, CEA and CA153 levels between patients with and without CSV-CTCs were found (*P*= 0.0063, 0.0191 and 0.0067, respectively). Significant differences of serum levels of CEA, CA125 and CA153 were found between CSV-CTC positive and negative patients (*P*= 0.0202, 0.0315 and 0.0279, respectively). Among the 4 serum tumor markers, CA153 is the tumor marker most related with LC patients’ clinicopathological characteristics.

**Table 6:** Correlation of serum tumor biomarkers with LC patients’ clinicopathological characteristics.

Characteristics	n	NSE (ng/mL)		*P*	CEA (ng/mL)		*P*	CA125 (U/mL)		*P*	CA153 (U/mL)		P
		Mean	Median		Mean	Median		Mean	Median		Mean	Median	
***Gender***													
Male	40	18.51	16.86	0.3019	28.48	4.04	0.4702	48.12	20.1	0.9643	23.37	12.19	0.4024
Female	27	23.24	18.2		111.65	3.08	55.08	17.74	30.56	14.28			
***Age***													
≥60	36	21.07	18.05	0.0518	94.66	4.47	0.3392	58.33	25.25	0.2473	31.1	16.75	**0.0291**
<60	31	19.06	14.71		24.06	2.59	42.31	16.58	20.65	10.04			
***Histology***													
Adenocarcinoma	56	20.41	17.05	0.6816	71.22	4.47	0.2395	55.88	18.44	0.9393	29.44	14.99	**0.0081**
Squamous	11	19.5	18.94		15.03	2.43	25.68	16.58	10.12	9.23			
***Smoking History***													
Yes	30	20.16	17.33	0.3993	28.28	3.965	0.6959	42.85	20.92	0.8352	20.02	11.44	0.2435
No	37	20.3	17.17		89.33	3.87	57.46	17.74	31.33	14.78			
***TNM Stage***													
I	21	13.59	13.37	**0.0018**	2.29	1.78	**<0.0001**	11	9.53	**<0.0001**	10.3	9.14	**<0.0001**
II	6	16.31	16.86		3.15	2.1	16.35	8.67	14.78	14.86			
III	13	19.81	18.94		41.34	3.81	48.39	21.07	17.12	11.33			
IV	27	24.92	20.17		131.45	11.4	91.26	40.68	45.64	27.24			
***Tumor Depth***													
T1	27	14.34	13.89	**0.0065**	107.9	2.53	0.052	18.66	13.16	**<0.0001**	14.34	10.99	**0.0024**
T2	11	20.24	18.52		7	3.81	18	11.68	15.83	11.55			
T3	16	20.18	17.19		44.84	5.175	45.71	29.4	24.59	16.71			
T4	13	27.98	19.06		34.26	10.08	152.2	96.68	61.94	33.53			
***Lymph Node Involvement***													
N0	30	14.86	14.71	**0.0033**	92.16	1.94	**0.0011**	17.39	9.995	**0.0002**	13.56	11.23	**0.0335**
N1	4	27.94	27.26		9.063	8.95	32.23	27.35	35.67	25.22			
N2	19	22.87	18.25		36.64	4.11	90.72	29	32.65	12.08			
N3	14	23.07	18.14		46.89	21.87	74.11	68.26	42.14	16.94			
***Lymph Node Metastasis***													
Yes	37	23.55	19	**0.001**	37.54	8.7	**0.0002**	78.11	29.79	**<0.0001**	36.57	16.9	**0.008**
No	30	14.86	14.71		92.16	1.94	17.39	9.995	13.56	11.23			
***Distant Metastasis***													
M0	40	16.32	15.85	**0.0046**	15.11	2.455	**<0.0001**	23.7	11.68	**<0.0001**	13.19	11.07	**< 0.0001**
M1	27	24.92	20.17		131.5	11.4	91.26	40.68	45.64	27.24			
***CSV Positive CTC Status***													
No Detected (0)	9	13.34	11.64	**0.0063**	2.38	2.31	**0.0191**	20.23	14.89	0.1981	9.532	8.73	**0.0067**
Detected (≥ 1)	58	21.4	17.8		71.25	4.47	55.68	22.65	28.86	14.53			
Positive (≥ 2)	40	21.55	17.18	0.2013	29.54	4.89	**0.0202**	62.52	25.27	**0.0315**	31.6	15.2	**0.0279**
Negative (< 2)	27	15.98	15.75		138.3	2.395	23.66	13.29	13.75	9.91			

LC, lung cancer; n, number; NSE, neuron-specific enolase; CEA, carcinoembryonic antigen; CA125/153, carbohydrate antigen 125/153; TNM, tumor-node-metastasis; CSV, cell surface vimentin; CTC, circulating tumor cell. Bold values mean statistically significant. Bold values are all less than 0.05.

ROC curves were drawn to compare the diagnostic performance of CSV-CTCs with serum tumor makers. The diagnostic efficacy of CSV-CTCs (AUC = 0.909) was significantly higher than those of NSE, CEA, CA125 and CA153 (AUC = 0.661, 0.673, 0.53, and 0.624, respectively, **Figures 3E–H**). Combinations of CTCs with serum tumor markers for diagnosing LC were also explored. However, the combination did not improve the diagnostic potency, for all AUC of combinations were reduced as compared to CTCs alone (**Figures 3E–H**).

## Discussion

Studies have suggested the use of CTCs for LC screening (15–17, 19). However, the low detection rate restricts the clinical application of CTCs as a diagnostic aid in practice. CellSearch^®^, the only CTC system that has been cleared by the US Food and Drug Administration (FDA) for clinical use in patients with metastatic breast, colorectal and prostate cancer (20–22), utilizes anti-EpCAM (epithelial cell adhesion molecule) immunomagnetic beads to capture epithelial CTCs. Krebs et al. used CellSearch^®^ to detect CTCs in stage III and IV LC patients, and the CTC detection rates were only 5% and 32%, respectively (23). Marchetti et al. used CellSearch^®^ and CTCs were detected in 15 out of 41 NSCLC patients (41%) (24). Ilie et al. used CellSearch^®^ and ISET^®^ to detect CTCs in advanced NSCLC patients, and the CTC detection rates were 32% and 76%, respectively (25). Many platforms have been developed to isolate CTCs, depending on either the unique biophysical or biochemical properties of CTCs, or a combination of both (26). Different CTC enrichment methodologies have different sensitivities for CTC detection. ISET^®^ stands for ^“^Isolation of Epithelial Tumour Cells by Size”, using microfiltration to enrich CTCs and then immunofluorescent staining of epithelial markers to identify CTCs. ISET^®^ usually has a higher CTC detection rate in LC than CellSearch^®^. Guibert et al. used ISET^®^, and CTCs were detected in 89 out of 96 advanced NSCLC patients (93%) (27). With the improvement of technologies, new CTC detection methods become more sensitive. Tong et al. used Cyttel, a negative immunomagnetic selection method, to detect CTCs in 127 patients with advanced NSCLC and the CTC detection rate was 84% (28). CytoSorter^®^, a microfluidic-based CTC immuno-capture platform, was employed in this study for CTC detection. One advantage about CytoSorter^®^ is that any biotin-labeled antibody can be immobilized on the streptavidin-functionalized CytoSorter^®^ nanochip for capturing desired cells. Epithelial‐to‐mesenchymal transition (EMT) is a common phenomenon during cancer development (29). EMT causes the reduced expression of epithelial markers, such as EpCAM and cytokeratin, and enhanced expression of mesenchymal markers, such as vimentin, twist, snail and slug (30). Over-expression of vimentin in cancer cells is highly correlated with cancer progression, and EMT would lead to the translocation of vimentin from the intracellular region to the cell surface to become cell surface vimentin (CSV) (31). It is reported that CSV can be used as a target for capturing EMT and mesenchymal CTCs (32). Previous study has shown CTC detection rate with CSV antibody was higher than that with EpCAM antibody in breast and pancreatic cancers (18, 33). This study aimed to evaluate CSV-CTCs as a biomarker for LC. Our first result indicate CSV-CTCs can distinguish LC patients from BLD patients and HD. As suggested by the other studies and our results, CTCs can be used in general as a diagnostic biomarker for LC (19).

When CTC cut-off was set to 2 CTCs per 7.5 mL of blood, CSV-CTCs have a sensitivity and specificity of 0.67 and 0.87, respectively, for diagnosing LC. The cut-off value is consistent with the previous finding in pancreatic cancer (18). Different CTC systems use different methodologies and thus have different sensitivities. Results from different CTC platforms are not comparable due to the different cut-off values caused by different sensitivities. **Table 7** summarizes recent studies concerning the use of CTCs as a diagnostic tool for LC (15–17, 34–37). In spite of different CTC detection methods, these studies all come to the same conclusion that CTCs can be used as a diagnostic biomarker for LC. Among them, EpCAM based methods, such as CellSearch^®^ or CellCollector, usually have a lower sensitivity due to the lower CTC detection rate. CSV-CTCs are usually CTCs that underwent EMT, representing the mesenchymal and the mixed types of CTCs. Our results show that CSV-CTCs can be used as a diagnostic biomarker for LC. As shown in **Table 7**, the diagnostic potency of the CSV based strategy is slightly better than the EpCAM based methods. Techniques using physical properties such as size difference or negative immuno-selection to enrich CTCs might have a higher CTC detection rate although with lower purity. High expression of FR alpha (FRα) is usually observed in LC, especially in adenocarcinoma. Therefore, FR based PCR has been proposed to detect CTC in LC, and the results are promising. FR based PCR method has a better sensitivity and specificity as shown in **Table 7** (16, 35). In fact, in the Chinese expert consensus on lung cancer screening and management, it is suggested that FR PCR based CTC detection can be used in conjugation with medical imaging examination to enhance the diagnostic specificity of lung nodule diagnosis (38). Li et al. used immunolipid magnetic spheres conjugated with 3 different antibodies, epidermal growth factor receptor (EGFR), vimentin and folic acid (FA), to detect CTC in early stage NSCLC patients. Using 2 CTCs per 7.5 mL of blood as cut-off value, the positive rates of EGFR, vimentin and FA magnets used alone and in combination in LC patients were 65%, 33.3%, 93.3% and 100%, respectively (39). It reconfirms that vimentin is not a good target to be used to capture CTCs in early stage LC. However, the combination of multiple targets, such as EpCAM, CSV, EGFR or FA, might be a good strategy to increase the CTC detection rate in early stage LC.

**Table 7:** Summary of studies concerning the diagnostic performance of CTCs in LC diagnosis.

First author	Year	Methodology	No of patients (control)	Mean age (year)	Cut-off value	AUC	Sensitivity	Specificity
Tanaka F (34)	2009	CellSearch^®^	125 (25)	N/A	CTCs ≥ 1/7.5 mL	0.598	0.3	0.88
Yu Y (35)	2013	FR PCR	153 (113)	59.4	8.64 CTC units	0.823	0.73	0.84
Chen YY (36)	2014	CD45 negative selection	50 (24)	59	CTCs ≥ 2/3.2 mL	0.917	0.84	0.98
Fiorelli A (37)	2015	ScreenCell (size)	60 (17)	65.5	CTCs ≥ 25	N/A	0.89	1
Chen X (24)	2015	CytoploRare (FR PCR)	473 (283)	55.1	8.93 CTC units	0.815	0.74	0.87
Li Y (25)	2019	NE‐FISH	174 (90)	N/A	CTCs ≥ 2/3.2 mL	0.846	0.68	1
Duan GC (23)	2020	GILUPI CellCollector	44(20)	56	CTCs ≥ 1	0.715	0.53	0.9
Our study	2020	CytoSorter^®^ CSV	78 (30)	57.2	CTCs ≥ 2/7.5 mL	0.767	0.67	0.87

CTC, circulating tumor cells; LC, lung cancer; no, number; AUC, area under curve; N/A, not applicable; FR PCR, folate receptor ligand-targeted polymerase chain reaction; CD45, cluster of differentiation 45; CSV, cell surface vimentin.

One major drawback of the CSV based method is that many false positives were found in patients with BLD. Although CSV positive cells were not detected in HD, they were found in 10 out of 21 patients with BLD. For patients with BLD who were found to have CSV positive cells, 90% of patients had either inflammation diseases, fibrosis or other lung infections. The reason for the false positive is that CSV is not a tumor specific marker. In fact, most of the targets used for CTC detection are not tumor specific. CSV has been identified to participate in cell adhesion, migration and cellular signaling (21). Expression of CSV is not only seen in cells undergoing EMT, but also in cells infected with certain virus, and in activated lymphocytes, myofibroblasts and stellate cells (40–42). As inflammation and fibrosis are usually common observed in patients with BLD, the false positives might come from the activated lymphocytes and myofibroblasts. A counterstain of activated lymphocytes and myofibroblasts or use of a tumor specific marker should be applied to reduce false positives.

CSV-CTCs are correlated with LC patients’ cancer stage, lymph node involvement and distant metastasis, which is consistent with previous findings that CTCs can reflect tumor burden in LC (15, 16). Our results surprisingly show that CSV-CTCs are correlated with LC patients’ age. It could be that older patents recruited in this study had usually advanced tumors. If LC patients were first grouped by cancer stage, no significant correlation between CTCs and age would be found in each group (data not shown). CSV-CTC enumeration was not associated with tumor size. CTCs captured by CSV antibody was only one subtype of CTCs. As Li used 3 different antibodies to enrich CTC in LC and found that only CTC enumeration detected by the combined use of 3 antibodies was correlated with cancer stages (39). It suggests that the total CTCs, but not one subtype, may be correlated with tumor size. However, CSV-CTC positive rate was correlated with tumor size, lymph node involvement, distant metastasis, but not with cancer stage, which might be explained by the small sample size. It is believed that CTCs undergoing EMT survive better in circulation and have higher chance to colonize at distant site to form metastasis (43). Therefore, cancer patients with metastasis should have more EMT and mesenchymal CTCs, which is in line with our findings in this study that LC patients with lymph node or distant metastasis tend to have more CSV-CTCs, which might represent the CTC subtype with more invasive nature.

Serum tumor markers have been used extensively in daily practice for LC diagnosis. Therefore, lastly, we liked to compare the diagnostic potency of serum tumor markers and CTCs for LC and see whether the combination of serum tumor markers with CTCs would improve the diagnostic performance. Serum levels of NSE, CEA, CA125 or CA153 cannot distinguish LC patients from the control, indicating that serum tumor markers are not reliable markers for LC screening. Similar to the CSV-CTCs, serum tumor marker test generates many false positives in patients with BLD. However, in some extent, all serum tumor markers can somehow reflect LC patients’ clinicopathological characteristics as shown in **Table 6**. Some serum tumor markers can even reflect the status of CSV-CTCs. Studies showed that a combination of several tumor markers can produce a higher sensitivity (44, 45). However, the best combination of tumor markers for diagnosing LC remains unknown. The AUC of the combined use of NSE, CEA, CA125 and CA153 is still smaller than that of CSV-CTCs alone (data not shown). Li et al. also found that the diagnostic sensitivity for LC yielded from the combination of four serum tumor markers, CEA, CA125, cytokeratin fragment 21-1 (CYFRA 21-1), and squamous cell carcinoma (SCC), was still lower than that achieved based on CTC counts alone (17). Conjugation of serum tumor markers with CSV-CTCs unexpectedly did not improve the diagnostic performance, which is contradictory to a previous finding that diagnostic performance for LC would be improved by combining CTCs with serum tumor markers (17). It could be explained by the different CTC populations captured in these two studies. While we detected the EMT CTCs, the other study detected total CTCs. Both CTC and serum tumor markers can generate false positive or negative result. Thus, they can only be used as a reference in practice. Biopsy should be still considered as the gold standard for disease confirmation.

Although it is reported that CSV can be used as a target to enrich CTCs in sarcoma, breast, pancreatic, prostate and gastric cancers (18, 32, 33, 46, 47), the high false positive results in patients with BLD raised the question that whether CTCs captured by anti-CSV were truly tumor cells. One major limitation of this study is that we did not collect the captured CTCs for any downstream analysis to confirm its identity. Also sample size was limited. We might get more statistically significant results if more LC patients with different cancer stages were recruited in this study.

## Conclusions

Results of this study show that CSV-CTCs can be used as an acceptable biomarker for LC with a sensitivity and specificity of 0.67 and 0.87, respectively. CSV-CTCs are positively correlated with lymph node and distant metastases, indicating that CSV-CTCs represent the CTC subtype with more invasive nature. Still further research with larger patient population is needed to verify our findings.

## Data Availability Statement

The raw data supporting the conclusions of this article will be made available by the authors, without undue reservation.

## Ethics Statement

This study followed the principles established in the Declaration of Helsinki and was approved by the ethics committee of the First Affiliated Hospital of Guangzhou Medical University. Written informed consent to the publication of their case details was obtained from each patient and healthy donor. The patients/participants provided their written informed consent to participate in this study.

## Author Contributions

Conception and design: XX, LW, XN W, SL and CZ. Acquisition of data: XX, LQ W, XN W, YQ, XL, ZX, ML, and MO. Analysis and interpretation of data: XX, LW, XW, W-HF, and CZ Z. Writing of the manuscript: XX and W-HF. All authors contributed to the article and approved the submitted version.

## Funding

This study is supported by the following grants: Zhongnanshan Medical Foundation of Guangdong Province [ZNSA-2020003], Guangdong Science and Technology Program special projects [2020A1111350025], Wu Jieping Medical Foundation [320.6750.19088-8], and Beijing Bethune Charitable Foundation [BQE-TY-SSPC(5)-S-03].

## Conflict of Interest

W-HF is currently an employee of Watson Biotech.

The remaining authors declare that the research was conducted in the absence of any commercial or financial relationships that could be construed as a potential conflict of interest.
